# A qualitative interview study of GPs’ experiences of prescribing opioid medication for chronic pain

**DOI:** 10.3399/BJGPO.2022.0085

**Published:** 2022-11-16

**Authors:** Simon Gill, John Bailey, Sadia Nafees, Rob Poole

**Affiliations:** 1 Wrexham Maelor Hospital, Betsi Cadwaladr University Health Board, Wrexham, United Kingdom; 2 Centre for Mental Health and Society, Bangor University, Bangor, United Kingdom

**Keywords:** analgesics, opioid, chronic pain, qualitative research, drug prescriptions, primary health care, general practice

## Abstract

**Background:**

Prescribing of opioid medication has increased over the past 20 years. Most occurs in primary care for chronic pain. There is little evidence that these drugs are effective for this indication. There are concerns about the continuing prescribing of opioids, particularly in the long term and at high doses.

**Aim:**

To explore GPs’ experiences of prescribing opioids, problems encountered, and factors militating against good prescribing practice.

**Design & setting:**

Qualitative interviews with GPs who prescribe opioids in primary care in North East Wales.

**Method:**

Semi-structured interviews with 20 GPs were transcribed and subjected to thematic analysis utilising the framework approach.

**Results:**

Participating GPs identified a range of problems associated with prescribed opioids. They were concerned about limited effectiveness of the drugs and what they perceived as addiction resulting from their use. They identified healthcare system factors that were obstacles to good prescribing practice such as lack of continuity of care, poor access to secondary care pain management support, and, most importantly, constant time pressure. They reported adverse effects on relationships with patients. Unrealistic expectations that pain could be eliminated resulted in pressure to prescribe stronger drugs and increased doses. It led to difficulties in establishing and maintaining trust and in persuading patients to agree to, and to carry out, dose reductions.

**Conclusion:**

Themes emerging from this study suggest that GPs lack appropriate control of opioid prescribing. There is a need to develop methods to help patients and GPs to work together to manage chronic pain safely.

## How this fits in

Opioid medication is frequently prescribed in primary care but is known to be largely ineffective in the long-term treatment of chronic pain. This study found that GPs are concerned by this situation and experience pressures to initiate, continue, or increase opioids against their better judgement. The factors they identified that make appropriate prescribing difficult to achieve must be addressed if long-term opioid prescribing is to be reduced.

## Introduction

There has been a large increase in prescribing of opioids in the UK since 1990.^1–3^ Prescribed opioids are an issue of widespread concern in the UK, with focus on avoiding the severe problems encountered in the US.^4,5^ Most opioid prescribing is for chronic non-cancer pain (CNCP)^6–8^ despite a lack of evidence of effectiveness for this indication^9,10^ and evidence of the risk of harms from using these drugs.^10–12^ There is epidemiological evidence that shows opioids for CNCP are associated with worse functioning and quality of life,^13–16^ and clinical experience suggests that this is particularly prevalent in patients on high doses.

Although overall opioid prescribing may have begun to decrease, rates are still much higher than they were 25 years ago.^2,7,17^ Of greater concern is the increased prescribing of stronger opioids and higher doses,^2,8,18,19^ and the increase in numbers of people prescribed these drugs long term,^17,20^ which might be overlooked in a downturn in total numbers of prescriptions or items issued. Not enough is known about patients who are prescribed high doses of opioids long term. They may gain no benefit from their medication, while being at significant risk of harm.

Studies of opioid prescribing in the UK published to date have reported aggregated data including the following: the number of prescriptions; the number of individuals receiving prescriptions; and total quantities prescribed at a population level. There is a need to examine prescribing at the individual patient level.^21^ In addition, little is known about the reasons for continued high levels of opioid prescribing. Previous studies have shown that conversations with patients about opioids are experienced by GPs as difficult and a cause of conflict.^22–24^ There is a need for a better understanding of the prescribing of opioid medication from the perspective of GPs.

The aim of this study was to explore GPs’ experiences of prescribing opioids in primary care, problems they encountered, and factors militating against good prescribing practice.

## Method

### Design and setting

The qualitative face-to-face interview study was conducted in North East Wales.

### Participants and recruitment

Participants were GPs who worked at least one session per week in primary care. With an aim to recruit 20 participants, 167 GPs from 39 practices were invited to participate via email in October 2019. (One GP practice was excluded because its staff had participated in a previous study^21^ related to the current research.) Two reminder emails were sent at 2-week intervals with a follow-up telephone call to practice managers after a further 2 weeks. Recruitment was completed in January 2020. Potential participants received an information sheet that included the following information:

There has been a large increase in the number of prescriptions for opioid drugs in the UK over the last 20 years, with most prescribing for persistent non-cancer pain. Recent research shows the increase in prescribing is continuing particularly for strong opioids. There is a lack of good-quality evidence that these medications are effective in managing persistent pain or improving function in the long term, or at high doses.

### Data collection

Face-to-face interviews in GP practices or other NHS premises were conducted by SG, a pharmacist working in primary care and an experienced clinical interviewer. He had previously met some of the interviewees through his routine clinical work. Interviews were conducted using the semi-structured topic guide (see Supplementary Table S1), which was based on the study aims and objectives, and refined by the study team (RP, JB, SN). The interviews began with a framing statement and an open-ended question asking GP participants to talk about their experience of opioid prescribing. The prompts and probes in the topic guide helped elicit further information relevant to the study aims.

### Qualitative analysis

All interviews were audio-recorded with consent and transcribed verbatim. Interpretative analysis was carried out, following the five stages of the framework approach, described by Ritchie and Spencer, and Gale *et al*,^25,26^ using Microsoft Excel. All transcriptions were read by SG and JB, who agreed to a preliminary coding, which was applied to three transcripts independently. The results were compared and a meeting with the wider group refined the coding, which was then applied to rest of the data by SG. Further interpretation workshops involving senior research team (RP, JB, SN) refined and agreed to the final themes (see Supplementary Table 2).

## Results

Twenty face-to-face interviews (8–31 minutes long) of GP participants, from 18 different practices, were completed from November 2019–January 2020. It was agreed by the team that data saturation had been achieved.

Following the framework approach, the following five main themes emerged from the analysis:

- General experiences of prescribing opioids
- Factors affecting good prescribing practice
- GPs’ interactions with their patients
- Lack of control
- Expert support to GPs.

### General experiences of prescribing opioids

#### Concerns

Most GP participants reported that they had misgivings about opioid medication use in primary care and that they had patients in their practice whose opioid use caused them concern. They identified these drugs as being associated with a wide range of problems. They frequently referred to very long-term prescribing of opioids; prescription of opioids alongside other sedative drugs, gabapentinoids and diazepam being specifically mentioned; negative side effects, particularly in long-term use; patients being prescribed high doses; and the lack of alternative interventions for pain. Other issues identified included the following: the use of pain medication to treat a different problem such as relieving anxiety; opioid-induced hyperalgesia; risks to older patients; inappropriate prescription for conditions such as fibromyalgia; and a culture of reliance on drug interventions. The GP participants frequently reported doubts about aspects of their current prescribing practice and some described a reluctance to initiate or increase opioid doses:

*'For many years I’ve worried about the over-usage of opioids ... They have effects above and beyond any potential painkilling effect.* […] *We know these drugs don’t really help very much, can make things worse, but it’s a difficult case to argue.'* (GP13)

#### Addiction

Some of the GP participants identified addiction as a major problem associated with prescribed opioids. They were aware of an increase in use in recent years and some referred to the consequences of overprescribing in the US. The intrinsically addictive nature of the drugs was cited; the problems encountered with patients prescribed them were ascribed to addiction. Patient requests, particularly when repeated, to increase doses, and the converse reluctance to decrease doses when advised, were often cited as evidence of addiction, reinforcing the need to control doses:

*'Slipping through the net are the drug-seekers, the addicts, the addicts with intention, or no intention.'* (GP19)

*'I think you can't start stripping people of medication that they’ve become dependent on.'* (GP14)

*'I think because the patients demand medication it would seem likely that we’re no longer treating pain, we are treating an addiction, basically.'* (GP15)

#### Ineffectiveness

The GP participants were concerned by the observed ineffectiveness of opioids; that long-term use often did not reduce pain symptoms. Many spoke about the limits to effectiveness, with benefits waning over time, and some identified a ceiling effect, whereby there was a point when increased doses brought no increase in benefits. They were also concerned about the negative effects experienced by patients when their drugs ceased to provide benefits and about the lack of available alternatives to opioids:

*'… realising that actually, they don’t get better. They don’t improve symptom-wise.'* (GP4)

*'... my understanding is that the … it’s very rarely effective to increase or escalate medication if it’s not working.'* (GP15)

*'When the family realises that, actually, the higher doses are not helping that much and they may be contributing to side effects.'* (GP9)

#### Patients who do well on high-dose opioids

GP participants were specifically asked if they were aware of any of their patients who were using high doses of opioids and who experienced good levels of pain relief without reduced levels of functioning and impaired quality of life. None of them were able to provide instances of high-dose patients doing well. Several did not answer the question and several others answered a different question; for example, describing patients who were not on high doses or who had improved after reducing doses. Some drew a distinction between doing well and being content with their opioid regimen.

### Factors affecting good prescribing practice

#### Time pressures

There were frequent references by most GP participants to the effects on prescribing practice of working within a system that creates constant time pressure. Time-limited appointments were cited as a barrier to dealing with complex problems and difficult-to-treat patients. They reported not having sufficient time for good management and monitoring of patients. Staff shortages exacerbated these difficulties, some GPs reporting having time to do little more than deal with immediate problems. It was suggested that because of time pressures simple options, such as repeatedly prescribing drugs, were more likely to be taken than the exploration of alternatives:

*'… right now, because we’re barely able to provide a basic appointment at the moment, we don’t really have any appointments to bring them for repeated review.'* (GP16)

*'I think the problem with the way it works in clinical practice is that it’s very hard to keep track of your patients. There are so many people on opioids and so many other issues that you present with that you don’t have the time to monitor.'* (GP2)

*'So, you will have the 10 minutes to address the main problem and the pain that comes as a result of it, which is a difficult and tricky one. So, I think it does feel, a lot of times, that the easiest option is to escalate whatever painkillers they're on. That would work, at least psychologically, to some extent.'* (GP19)

#### System constraints

Most GP participants reported a range of constraints, associated with a fragmented healthcare system, which affected the ability to deliver good practice, either in general or in relation to the treatment of pain and the prescribing of opioids. Negative external factors were reported including the following: the influence of the pharmaceutical industry, past and present; policy directives; and the difficulties in accessing secondary care services and, in particular, long waiting times for those services. There were references to patients discharged from secondary care on drugs and doses that were difficult to discontinue or reduce. This was also true of patients in primary care who were already on high doses when either inherited from their previous GP or transferred from another practice. Lack of continuity of care within practices, with patients being treated by several clinicians, was seen as making consistent strategies for managing prescribing difficult and tending to facilitate increasing doses:

*'I trained as a junior doctor, a house officer from 2008. At that time opioids were actually being promoted for non-cancer pain.'* (GP4)

*'Regarding the opioids, the challenge I find is that these patients, often in modern primary care they are seen by various different clinicians. It’s probably easier to give in when we don't see the patient in continuity, right?'* (GP9)

*'My own experience of being a GP for about 20 years, I’ve come across more patients on opiates from secondary care and other GPs than I’ve started.'* (GP20)

*'… we inherit sometimes patients here that come from other surgeries or historically, have been on significant amounts of opioids for non-cancer pain.'* (GP11)

*'We keep coming back to higher strength opioids. In the absence of quick access to physiotherapy, in the absence of quick access also to a pain clinic and long appointment waits and so on, it keeps coming back to a medication solution. That’s what patients tend to expect.'* (GP13)

### GPs’ interactions with their patients

#### Patient expectations

A factor that GP participants reported as creating difficulties was patients’ beliefs and expectations about pain and the drugs they take for pain. They cited patient expectations that chronic pain could be removed completely; that this could be achieved through drugs; and, if this was not currently happening, it would be achieved through increased doses or stronger drugs. They described difficulty in persuading patients that these expectations could not be fulfilled. They reported that this led to continual pressure for increased doses, different drugs, early prescriptions, and referral to specialist services. The converse of this was a reluctance to decrease doses of drugs that were ineffective or had negative effects, which was frequently driven by the fear that reductions would lead to intolerable worsening of pain:

*' … it is difficult, pain relief, I think, because patients expect you to give you something to make it completely pain free, something that is safe, that doesn’t give them side effects, and the fact that there isn't something like that, it’s hard for them to understand, isn't it?'* (GP11)

*'We get a lot of requests to increase it, because there is this sort of, I guess, lay belief that the more you take, the more likely it is to work.'* (GP12)

*'You’re constantly being under pressure, “The painkiller doesn’t work, have you got something different? Have you got something stronger?”'* (GP2)

*'It is very difficult in some patients to even talk about trying to reduce the medication so that the medication then becomes sort of patient demanded or patient-led.'* (GP15)

*'It provokes enormous anxiety. I think I would say that was the … First of all it’s, kind of, “You’re trying to take something away from me,” but the impression that you get from the patient is extreme anxiety that they’re going to be in pain that they cannot then address.'* (GP17)

#### Relationship

In dealing with the complex issues around the prescribing of opioids for chronic pain, GP participants described the importance of building good relationships with patients. There were references to the skill, effort, and, above all, time needed to engage with patients to develop mutual trust, understanding, and agreement:

*'The starting point for me is establishing a really good, mutually trusting relationship with a patient, and then, once I've got them on side, I then start to explain to them the harmful effects of these drugs, and start to try and chip away at them.* […] *One of the first things I do is start to try and chip away, but that requires quite a lot of consultations to get to that point, quite a lot of explanation, but more importantly, it requires the skill of getting into them, inside them, and getting to the core of the problem.'* (GP14)

There was also recognition of the dangers of breakdown in relationship when engagement fails. Some spoke of a fear of damaging relationships by discussing opioid issues. For some, the need to maintain good relationships outweighed the need to address opioid-related problems. Some saw unmet expectations and relationship breakdown as potentially leading to patients leaving the practice or making formal complaints:

*'“Why are you going to take me off this medication?” it will be the first question they ask, and we have several patients, actually, coming from other surgeries because of their experience with the GPs taking them off the medications. So, this can result in poor patient–doctor relationship, can lead to complaints and so we’re on a negative feedback end result.'* (GP8)

#### Dose reduction

GP participants were specifically asked if they had found reducing or stopping opioids a successful strategy. The majority reported attempting this with a few patients but not making it a widespread practice. Many described the process as being difficult to initiate, with patients finding the concept of improvement through reduction of pain medication hard to accept. Persuading patients to begin and to continue reducing doses and monitoring progress placed large demands on time and resources. There were also reports of strained doctor—patient relationships. A few described successful outcomes with some patients but most reported little or no success. Some reported patients reaching a point where they refused to make further reductions, achieving only partial success.

### Lack of control

Throughout the interviews GPs described factors giving rise to long-term and high-dose opioid prescribing. A cross-cutting theme of a lack of control or agency emerged from many different and interconnected issues described by the GP participants. 'Addiction' referred to a situation where patients’ drug use was out of control. Opioid ineffectiveness, even in high doses, meant that the symptom of pain could not be controlled. Working under constant time pressures meant that best clinical practice was hard to deliver and GP participants lacked the control to give sufficient time to patients with complex and difficult problems associated with chronic pain and medication use. A lack of continuity of care, particularly between prescribers, made control of an overall clinical strategy hard to achieve. Pressures to meet patient expectations and a fear of damaging relationships inhibited attempts to control medication use. Many GP participants found establishing control by systematically reducing doses was difficult to initiate and carry out successfully. Lack of timely access to other specialist services reinforced lack of control.

Many GP participants acknowledged, as a consequence of control issues, it was sometimes easier to prescribe opioids, increase doses, change to stronger drugs, or perpetuate regimens, despite many citing that long-term opioids did not work for chronic pain, and none being able to recall patients on such doses 'doing well'.

### Expert support to GPs

#### Available assistance

GP participants identified three services they currently drew on for support with prescribed pain medication, these were as follows: NHS primary care pharmacists; secondary care pain management teams (PMT); and the Prescribed Medication Support Service (PMSS). (PMSS is an NHS counselling and support service for patients prescribed dependency forming medicines, including opioids, which is unique to North Wales [see Supplementary Box 1]). Comments about pharmacists and PMSS were positive. Comments about PMTs were mixed, with long waiting lists drawing particularly negative comment.

#### Future needs

All GP participants made suggestions for future assistance in managing opioid prescribing. These fell into the following four categories:

1. Provision of resource materials for patients, which consists of education and information materials about the limitations of opioids.
2. Introduction of new or improved services, which includes the following: provision of improved psychology services; establishment of patient support groups; more primary care pharmacist support; direct GP access to PMT expertise for advice; earlier patient access to PMT; and a substance misuse-like service.
3. Wide-scale NHS organisational changes, which means greater consistency between primary and secondary care, prescribing protocols, drug formulary changes, and regulation of prescribing.
4. Better pain management training and education for GPs and healthcare workers, and provision of resource materials.

## Discussion

### Summary

The following five main themes emerged from the analysis: general experience of prescribing opioids; factors affecting good prescribing practice; GPs' interactions with their patients; lack of control; and expert support to GPs. The findings have shown that GPs are concerned about opioid prescribing in primary care. They often feel that they are presiding over unsatisfactory opioid regimens, but that there are major obstacles to reducing opioid use among their patients. These obstacles fall broadly into the following three categories: patient factors, such as the perception of addiction or unrealistic expectations of complete relief of chronic pain; system factors, such as time pressures and long waiting lists for secondary care pain management services; and interpersonal factors, such as fear of causing a breakdown in therapeutic relationships or provoking complaints. The consequence is a sense of lack of control over the patient's medication use and their pain management.

GP participants’ suggestions to improve the situation broadly match these categories, which are as follows: measures to change patient expectations such as patient educational materials; better and more responsive secondary care services; organisational changes, including greater external controls on their prescribing; and better training for primary care workers.

### Strengths and limitations

The study used a well-established qualitative method that allowed free exploration of GP experiences and attitudes without the research team making prior assumptions that might influence findings. The strength of the method is that it can result in novel and unexpected findings in areas such as opioid prescribing where there are intractable problems in achieving desired change (that is, reduction in dysfunctional and ineffective prescribing regimens).

All qualitative studies are vulnerable to the criticism that findings only apply to a particular small group of participants at a particular time. A potential weakness of this study is that participants were volunteers who responded to a general invitation to take part. They may, therefore, have had particular interests in or concerns about the topic, which made them unrepresentative. The number of participants in qualitative studies is typically small, and in this case 20, but analysis of interview content is detailed. It is necessary to be cautious when generalising findings but set against this, the data are rich in detail of GP experiences. The themes that have emerged in the study are familiar to GPs and pain medicine professionals. The main strength of the study is that the findings suggest some strategies to improve opioid prescribing in primary care that can be developed and tested in clinical trials.

### Comparison with existing literature

To the best of the authors' knowledge, this is the first study to focus directly on UK GPs' experiences of and attitudes to prescribing opioids for chronic pain. The most important finding is that the GP participants appear to have felt an uncomfortable lack of agency over opioid prescribing, with ambiguity whether the locus of control of opioid prescribing lies with doctor, patient, or neither. A qualitative study with UK GPs and patients,^23^ aimed at understanding processes leading to long-term prescribing of opioids, reported similar ambiguities. This was seen as resulting from poor access to specialist services and a lack of alternatives to medication as treatment options. Systems that did not facilitate continuity of patient care were also seen as leading to lack of control over prescribing. Discussions about dose reductions or patient requests for increases led to difficult conversations and sometimes conflict with patients. A Canadian interview study,^24^ with aims similar to the present study, showed that family practitioners (FPs) experience unease and uncertainty about opioid prescribing and have concerns about addiction and misuse. It is interesting that, in a different healthcare system, there were also problems around accessing and communicating with specialist services; inadequate provision of resources; doubts about FPs’ capabilities; and inadequate guidance about prescribing for pain. FPs found dose tapering difficult to carry out because of high failure rates and resistance from patients. Again, this often led to difficult conversations and confrontation. As in the present study, there were participants in both these studies who admitted to agreeing to maintain or increase doses in order to avoid conflict with patients.

### Implications for research and practice

The findings suggest that GPs are likely to be amenable to novel interventions and strategies to reduce the number of patients on ineffective and/or harmful opioid regimens. In the authors' opinion, the GP participants were right to identify major systemic problems, such as insufficient continuity of care to allow pursuit of longer-term treatment strategies, and fragmentation of secondary care services with consequent dislocation from primary care. Unfortunately, these systemic problems are well-recognised and are unlikely to improve in the immediate future. However, the findings suggest some interventions that might be effective within primary care, including better information on pain management for patients and training for primary care staff. Helping GPs with 'difficult' interactions has been explored previously with respect to cancer care and successful interventions have been developed.^27^ Similar or adapted approaches may help GPs with interactions with patients in chronic pain, and such an intervention is now being developed by the authors.

## Data

It would be inappropriate to publish the data on which the conclusions of this research rely as this would be outside the terms of the informed consent to participate.
